# Does market segmentation hinder interregional CO_2_ flow in China? — Evidence from China’s interprovincial MRIO table

**DOI:** 10.1371/journal.pone.0255518

**Published:** 2021-08-02

**Authors:** Shuang Wu, Jialing Zou

**Affiliations:** 1 Research Institute for the Guangdong-Hong Kong-Macau Greater Bay Area, Guangdong University of Foreign Studies, Guangzhou, China; 2 Division of Humanities and Social Sciences, Beijing Normal University-Hong Kong Baptist University United International College, Zhuhai, China; 3 Guangdong Institute for International Strategies, Guangdong University of Foreign Studies, Guangzhou, China; China University of Mining and Technology, CHINA

## Abstract

China is the key player in the globalization era and is eliminating its intra-national trade barrier. This process will affect interprovincial CO_2_ flows. This study recalculates interprovincial CO_2_ flows in China by using the latest MRIO table and applies a gravity model to assess how market segmentation affects interprovincial CO_2_ flows. Results show that the total volume of interprovincial embodied CO_2_ flow did not increase excessively from 2007 to 2012, but the pattern of embodied CO_2_ flow had changed a lot. Market segmentation significantly decreased the interprovincial embodied CO_2_ flows in China and within its sub-regions. At interregional level, market segmentation’s negative effect was significant between Central and Western China. Other variables such as geographical distance showed a significant negative impact on interprovincial embodied CO_2_ flow in China. On the basis of our results, we raise some relevant policies to deal with the environmental inequality caused by the decrease in market segmentation.

## 1. Introduction

During China’s planned economy, the central government emphasized on planning, autarky, and provincial self-sufficiency, which resulted in a relatively independent fiscal system at the provincial level and a certain degree of market segmentation (i.e., set up entry barriers and resource outflow restrictions) [1]. This kind of domestic market segmentation was gradually eliminated as the transportation infrastructure developed and the whole economy grown since the reform and opening up in 1978. [2]. In recent decades, increasing attention has been paid to global climate change. China has become the world’s largest carbon emitter since 2006, playing an essential role in global carbon reduction. China is ambitiously determined to reduce its carbon intensity by 60%–65% in 2030 from the baseline of 2005 and peak its total carbon emission by 2030. As a vast country with 31 provincial-level administrative regions and nearly 1.4 billion people, China’s carbon emission reduction targets would be allocated to every province. Many studies have demonstrated the interregional carbon flows embodied in interregional trade. Such studies highlight that understanding the carbon flows pattern embodied in trade would contribute to the overall carbon reduction targets. Among these studies, we find limited research on how market fragmentation affected interregional carbon flow, which is crucial because it relates to China’s regional environment equality.

The assessment of the interregional CO_2_ flow embodied in trade in China has been widely concerned. Many methods have been developed, such as life cycle analysis (LCA), and ecological network analysis (ENA). The LCA method tracks one specific product or service’s carbon footprint along with its whole life cycle [3, 4]. It is clear and precise but could be complicated if applied to the macro-level because it requires many datasets. Furthermore, LCA limits, such as the system boundary’s truncation, could lead to confusion or misinterpretation [5]. ENA is a systematic method frequently used to simulate the material and energy flow within a network system [6, 7]. It uses nodes and flows to represent the components and connections within the ecosystem network. It is widely used to measure carbon flow at the city or sub-city level [8–10].

Another frequently used method is the input-output analysis (IOA). Incorporating the environmental extension, the IOA has been extensively used in estimating the interregional CO_2_ flows embodied in trade. It was applied in multi-levels, such as international level, inter-country level [11], and inter-city level [12]. Combining multiple single input-output tables, the multiregional input-output analysis (MRIO) is developed, allowing scholars to evaluate carbon emissions embodied in interregional intermediate products and trade final products flexibly. Using the MRIO table of China, Feng et al. [13] estimated China’s provincial CO_2_ flow, indicating that nearly 60% of China’s emissions are embodied in interprovincial traded goods. Mi et al. [14] further investigated China’s interregional CO2 flow pattern from 2007 to 2012, where specific embodied CO_2_ flows reversed after the financial crisis.

A few attempts have combined the MRIO and ENA methods to estimate the interregional CO_2_ flow. Duan et al. [15] integrated MRIO and ecological network analysis to estimate the interregional CO_2_ flows embodied in trade. The most important implication of such analysis is that this framework differentiates the production-based and consumption-based carbon emission, which is closely linked to a given region or economy’s responsibility. [16, 17]. Given that increasing attention has been paid to consumption-based accounting methods, Steininger et al. [18] argued that none of the accounting methods could be viewed as the best.

Understanding the driving forces of the changes of interregional CO_2_ flow would allow scholars to obtain possible implications for carbon reduction. Many studies have employed a structural decomposition analysis (SDA) to investigate the driving factors [14]. However, an SDA is limited to the functional form of a given model [19], which means we can hardly capture endogenous effects flexibly and consider various factors based on its framework. The econometric panel model will be an effective supplement to tackle this problem because other qualitative driving factors can be added. Rosa et al. [20] first combined the MRIO-gravity approach to investigate the driving factors of carbon flows and supply chains, including production and consumption perspectives, geographical factors, structural factors, and institutional factors.

Although China’s opening-up policy has been implemented for more than 40 years, an obvious trade barrier is still observed among provinces and mainly caused by local protectionism. The trade barrier among provinces in China is market segmentation leading to the high cost of interregional products and services trade. Thus, an interesting question follows. How would the market segmentation impact the interregional CO_2_ flow embodied in trade? A few studies have investigated the market integration effects on environmental issues. Li and Lin [21] indicated that regional integration in China is correlated to CO_2_ emissions positively. He et al. [22] investigated the relationship between regional integration and CO_2_ marginal abatement cost. They illustrated that regional integration increased the CO_2_ marginal abatement cost in China.

Using panel data of interprovincial embodied CO_2_ flow and market segmentation, in this study, we explore the following questions: Does market segmentation impact the interregional CO_2_ flow? How would this impact be reflected in the different regional level? By answering these questions, this paper makes contributions in the following three aspects: (1) Previous studies generally focus on the impact of market segmentation on commodity flow but lack research on virtual product flow. In this paper, we empirically analyze the impact of market segmentation on virtual carbon flow for the first time; (2) Based on the traditional gravity model, we reconstruct the estimation model of the embodied carbon flow considering the effect of market segmentation (3) According to the results of our analysis, we put forward policy implications for market reform and regional environmental equity.

The rest of paper is organized as follows: Section 2 will introduce the methodology and data. Section 3 will present the results of interprovincial embodied CO_2_ flows embodied in trade and the estimation results from the gravity model. Section 4 will compare our results to similar studies. Section 5 will note policy implications and conclude.

## 2. Methodology and data

### 2.1. Methodology

The main purpose of this paper is to explore the impact of market segmentation on inter provincial CO2 flow. We use a step-wise method to solve this problem. First, we employ the multiregional input-output (MRIO) model to calculate the interprovincial CO2 flow. According to Duan et al. [15], MRIO model is widely used in the calculation of inter-regional CO2 flow. Second, we calculate the degree of market segmentation among China’s provinces. Third, we estimate the impact of market segmentation on CO2 flow based on gravity model. The way CO2 flow is similar to commodity trade. Since gravity model is often used to estimate commodity flow, we refer to gravity model to estimate the CO2 flow in our study. The specific calculation is described as follows.

#### 2.1.1. Calculation of interregional CO_2_ flow

Environmental IOA (EE-IOA) extends from Leontief IOA. The MRIO analysis can be expressed as follows:

$$\left[\begin{array}{c} X^{1}\\ X^{2}\\ \vdots \\ X^{m} \end{array}\right]=\left[\begin{array}{cccc} A^{11} & A^{12} & \cdots & A^{1m}\\ A^{21} & A^{22} & \cdots & A^{2m}\\ \vdots & \vdots & \ddots & \vdots \\ A^{m1} & A^{m2} & \cdots & A^{mm} \end{array}\right]\left[\begin{array}{c} X^{1}\\ X^{2}\\ \vdots \\ X^{m} \end{array}\right]+\left[\begin{array}{c} \sum_{q}^{M}Y^{1q}\\ \sum_{q}^{M}Y^{2q}\\ \vdots \\ \sum_{q}^{M}Y^{mq} \end{array}\right],$$ (1)

where X^p^ represents the total output of region p (p = 1,2,…,m). Y^pq^ represents the final demand of region q (q = 1,2,…,M) for products from region p. A^pq^ is the direct input coefficient matrix, which expresses the intermediate input in region q of commodities produced in region p. The elements of the direct input coefficient matrix can be shown as ${\text{a}}^{\text{pq}}=u_{ij}^{pq}/x_{j}^{q}$, where $u_{ij}^{pq}\left(i,j=1,2,\cdots ,n\right)$ shows that the commodities of the sector i of region p assign to the sector j of region q. The direct input coefficient matrices satisfy U^pq^ = *A*^*pq*^, *X*^*q*^ Eq (1) can be rewritten as follows:

$$\left[\begin{array}{c} X^{1}\\ X^{2}\\ \vdots \\ X^{m} \end{array}\right]={\left[\begin{array}{cccc} I-A^{11} & -A^{12} & \cdots & -A^{1m}\\ -A^{21} & I-A^{22} & \cdots & -A^{2m}\\ \vdots & \vdots & \ddots & \vdots \\ -A^{m1} & -A^{m2} & \cdots & I-A^{mm} \end{array}\right]}^{-1}\left[\begin{array}{c} \sum_{r}^{M}Y^{1r}\\ \sum_{r}^{M}Y^{2r}\\ \vdots \\ \sum_{r}^{M}Y^{mr} \end{array}\right]=\left[\begin{array}{cccc} L^{11} & L^{12} & \cdots & L^{1m}\\ L^{21} & L^{22} & \cdots & L^{2m}\\ \vdots & \vdots & \ddots & \vdots \\ L^{m1} & L^{m2} & \cdots & L^{mm} \end{array}\right]\left[\begin{array}{c} \sum_{r}^{M}Y^{1r}\\ \sum_{r}^{M}Y^{2r}\\ \vdots \\ \sum_{r}^{M}Y^{mr} \end{array}\right],$$ (2)

where L^pq^ is Leontief inverse, indicating the amounts of total output from region p per one-unit increase in the final demand of region q. Through Eq (2), we can obtain Eq (3) regarding the embodied CO_2_ of consumption between regions p and q based on environmental IOA. Specifically, $\hat{c}$ is a diagonal matrix of direct emission intensity coefficients, representing the volume of CO_2_ emissions per unit of output. Instead of the final demand, we calculate the embodied CO_2_ emissions result from interregional consumptions based on Eq (3).


$$E=\hat{c}x=\hat{c}{\left(I-A\right)}^{-1}Y=\hat{c}LY$$ (3)

#### 2.1.2. Estimation of Chinese interprovincial market segmentation

Market segmentation reflects the barriers to the flow of goods between regions caused by local protectionism, manifesting as the price difference of the same product or service between two adjacent regions. Furthermore, the greater the differentiation in price, the more the market, and the less the market is integrated. We calculate the market segmentation index for each province in 2007, 2010, and 2012. We further select the price indexes of agricultural products, industrial products, construction products, and services. Data unavailability precludes us from obtaining the service price index directly. Thus, we choose the wage index of service employees as a proxy indicator. Because the production cost of most services mainly comes from the wage of employees. And the increase of service employees’ wage index always leads to the increase of services price.

Following Parsley and Wei [23], we first calculate the price differentiation index for product *k* between regions *i* and *j* at time *t*, which can be expressed as follows:

$$\Delta Q_{ijt}^{k}=lnp_{i,t}^{k}-lnp_{j,t}^{k},$$ (4)

where $\Delta Q_{ijt}^{k}$ is the common currency percentage price difference, $lnp_{i,t}^{k}$ represents the price of good *k* in region *i* at time *t*, and $lnp_{j,t}^{k}$ represents price of good *k* in region *j* at time *t*. *Qt* is the chain index of the price (p) of goods *k* in year *t*. We use the absolute value |*ΔQijt* | to measure the price dispersion.

China’s product price index usually uses a month-by-month format. Thus, we can use the first-order price difference to measure relative prices. To avoid the relative price being affected by order of different regions, we change it into an absolute value format and obtain the following:

$$\left|\Delta Q_{i,j,t}^{k}\right|=\left|ln\left(p_{i,t}^{k}/p_{i,t-1}^{k}\right)-ln\left(p_{j,t}^{k}/p_{j,t-1}^{k}\right)\right|.$$ (5)

Furthermore, to offset the price differentiation caused by the commodity’s characteristics, we use the standard deviation method proposed by Parsley and Wei [24]. We then can obtain the degree of market segmentation caused by a particular market system, which is shown as follows:

$$maketSeg\_od_{i,j,t}=\sqrt{\frac{\sum_{k=1}^{n}{\left(\left|\Delta Q_{i,j,t}^{k}\right|-u_{i,j,t}\right)}^{2}}{n-1}}\;\text{and}\;u_{i,j,t}=\frac{1}{n}\sum_{k=1}^{n}\left|\Delta Q_{i,j,t}^{k}\right|,$$ (6)

where *maketSeg*_*od*_*i*,*j*,*t*_ is the degree of market segmentation between regions *i* and *j* at time *t*.

#### 2.1.3. Effects of market segmentation on interregional CO_2_ flow

To estimate the interregional CO2 flows’ influencing factors, we develop the model based on the gravity model. Following the recommendations from previous studies, namely, Bergstrand [25], Feenstra et al. [26], Anderson and van Wincoop [27], and Rosa et al. [20], we set interregional CO_2_ flows as a dependent variable and population, GDP per capita, spatial distance, and a common border of regions *p* and *q* as control variables. Concerning the flow of CO_2_ emissions, we also added the energy consumption per capita as a control variable. It is because if the destination of CO_2_ flow has a high energy consumption per capita, the CO_2_ emissions may also be high. The degree of market fragmentation is added as an independent variable. The model can be described as follows:

$$\begin{array}{l} ln\left(CO_{2}\right)=\beta_{0}+\beta_{1}ln\left(Pop\_o\right)+\beta_{2}ln\left(Pop\_d\right)+\beta_{5}ln\left(Aenergy\_o\right)+\beta_{6}ln\left(Aenergy\_d\right)+\\ \beta_{7}ln\left(Marketdis\_od\right)+\beta_{8}ln\left(Distance\_od\right)+\beta_{9}Contiguity\_od. \end{array}$$ (7)

### 2.2. Data resources and manipulations

We estimate interregional CO_2_ flows by using the MRIO tables of China’s multiregional input-output table in 2007, 2010, and 2012, including 30 provinces of China. The emission coefficients of fossil fuels for calculating CO_2_ emissions are obtained from IPCC. The population, GDP per capita, and energy consumption statistics are collected from China Statistical Yearbook and China Energy Statistical Yearbook. The spatial distance between provinces is calculated by measuring the distance between the provincial geo-center. For the common border, spatial distance would be 1 for geographical adjacent between two regions and 0 otherwise. All data used in the model are deflated to the constant price in 2007.

## 3. Results

### 3.1. Interprovincial embodied CO_2_ flow of China from 2007 to 2012

**Fig 1:**
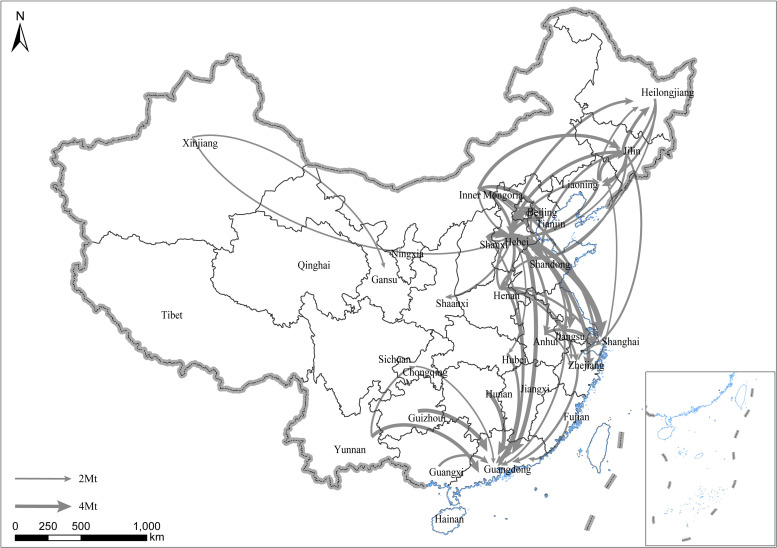
Interprovincial embodied CO_2_ flow of China in 2007. Only flows exceeding 2 Mt are included. The arrow indicates the direction of each flow. The width of the flows indicates the amount. Republished from standard map service of NGCC [28] under a CC BY license, with permission from Chinese National Basic Geographic Information Center, original copyright 2020. Note: The base map of Figs 1–3 is from the website "http://bzdt.ch.mnr.gov.cn/", which is run under National Geomatics Center of China (NGCC) for public use. It is an open access website in need of no permission for publication.

**Fig 2:**
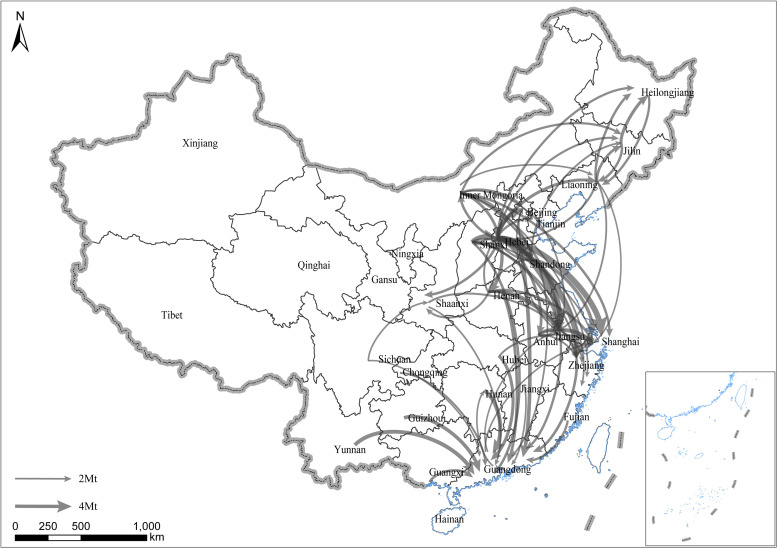
Interprovincial embodied CO_2_ flow of China in 2010. Only flows exceeding 2 Mt are included. The arrow indicates the direction of each flow. The width of the flows indicates the amount. Republished from standard map service of NGCC [28] under a CC BY license, with permission from Chinese National Basic Geographic Information Center, original copyright 2020.

**Fig 3:**
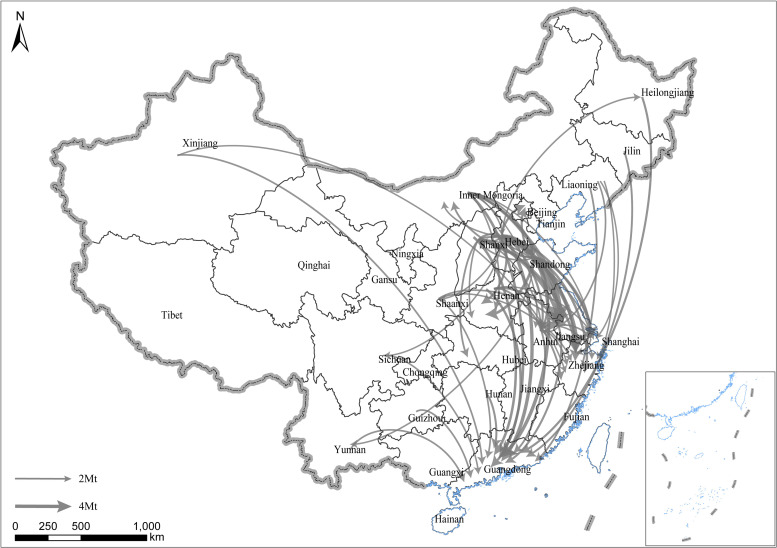
Interprovincial embodied CO_2_ flow of China in 2012. Only flows exceeding 2 Mt are included. The arrow indicates the direction of each flow. The width of the flows indicates the amount. Republished from standard map service of NGCC [28] under a CC BY license, with permission from Chinese National Basic Geographic Information Center, original copyright 2020.

We first calculated the interprovincial embodied CO_2_ flow of China from 2007 to 2012, and the results have been visualized from Figs 1–3. In Fig 1, the results show that the embodied CO_2_ flows out from rich energy provinces or provinces with heavy industries and mainly flows into China’s rich coastal provinces, such as Guangdong, Shanghai, and Zhejiang. In 2007, Hebei had the largest amount of domestic exporting embodied CO_2_, reaching over 80.0 Mt, which is followed by Henan (42.8 Mt) and Inner Mongolia (39.6 Mt). A large part of heavy industries, such as iron steel production, are clustered in these provinces. Inner Mongolia is known as the “Coal Capital” of China. In terms of CO_2_ inflow embodied in trade, Guangdong sees the largest amount by exceeding 63.8 Mt, followed by Shanghai and Zhejiang with 47.8 and 36.9 Mt of CO_2_, respectively. We also ranked the largest amount of embodied CO_2_ flows among provinces and find that the largest one is Hebei to Shanghai (6.7 Mt), followed by Shanxi to Hebei (5.9 Mt) and Hebei to Guangdong (5.5 Mt).

The pattern of interprovincial flow of embodied CO_2_ of 2010 is shown in Fig 2. The total flow of embodied CO_2_ is 728.1 Mt, which was 8.7% higher than that in 2007. The major flows were more concentrated in eastern and central provinces compared with that in 2007. The results show that the largest amount of embodied CO_2_ (domestic) exporting flows were also from Hebei (82.1 Mt), Henan (42.9 Mt), Shandong (40.7 Mt), Inner Mongolia (39.2 Mt), and Shanxi (36.3 Mt). Similarly, the largest embodied CO_2_ importers were Guangdong, followed by Shanghai, Jiangsu, and Zhejiang, but with a substantial increase compared with that in 2007. The top four flows all outflowed from Hebei province to Jiangsu, Shanghai, Zhejiang, and Beijing with volumes of 7.6, 7.6, 6.1, and 5.7 Mt, showing a diversified flow of embodied CO_2_ from Hebei. The fifth-largest flow was from Shanxi to Hebei with a volume of 5.4 Mt.

Fig 3. illustrated the major interprovincial flows of embodied CO_2_ in 2012. In Fig X, we find that the total flow of embodied CO_2_ only increased by 1.1% compared with 2010, which was much lower than China’s total GDP growth during the same period. The provinces that outflowed embodied CO_2_ were Hebei, Shandong, Shanxi, and Inner Mongolia with volumes of 69.9, 57.7, 44.7, and 41.3 Mt, which were not significantly different from those in 2010. Similarly, the provinces that inflowed the most were still Guangdong, Jiangsu, Zhejiang, and Shanghai. However, the pattern of the flows slightly changed, and the major flows became lesser in Northeast China than in 2007 and 2010, which was following the change of economic status in Northeast China. Furthermore, the top flows were outflows Hebei to Jiangsu, Zhejiang, and Guangdong with volumes of 5.9, 5.5, and 4.8 Mt. The following flows were outflows from Shandong to Guangdong and from Shanxi to Jiangsu province.

By looking into the change of embodied CO_2_ flow, we can find that the total flow volume has not increased significantly, but the pattern and volume of embodied CO_2_ flow have changed considerably during the study period. As indicated above, the pattern in China’s provincial CO_2_ flow embodied in trade has changed. We then explore whether market integration has played a role in such mode change. We will use econometric models in the next part to find further evidence.

We attribute the changes of interprovincial embodied CO_2_ flow in China from 2007 to 2012 to the following reasons. First, China experienced great macro-economic structural transformation during 2007–2012, which led to a narrowing regional disparity. Second, China has paid more and more attention to the problem of pollution transfer in general. The central government issued a series of policies on interprovincial ecological compensation, which stimulated the ecologically fragile provinces to protect their ecological environment. Third, China published the “National Main Functional Area Plan” in 2010 to set up ecologically fragile areas nationwide. The criteria of the (re)location of high energy consuming industries among provinces has become more stringent.

### 3.2. The effect of market segmentation on CO_2_ embodied flow result in China’s provincial consumption

#### 3.2.1 The effect of China’s interprovincial market segmentation impact on the CO_2_ embodied flow

To estimate the results of the model in Eq 7, we used the pooled OLS, fixed effects (FE), random effects (RE), GLS, and MLE models to estimate the global impact of interprovincial embodied CO_2_. The calculated interprovincial embodied CO_2_ flows did not have zero flows. Thus, we did not include the pseudo-maximum likelihood method. The regression results were reported in Table 1. As shown, the population effect of origin and destination had a positive effect on interprovincial embodied CO_2_ flows. The GDP per capita of the destination had a significant positive effect, whereas the GDP per capita of origin did not show a significant negative or positive effect in the time fixed model. The most critical issue is that the results show the market segmentation’s negative impact on interprovincial embodied CO_2_ flows in China and its decay with geographical distance.

**Table 1:** The gravity estimated results using China’s interprovincial embodied CO_2_ flows as dependent.

Variables	OLS	FE	FE-T	FE-TW	RE-GLS	RE-MLE
**Pop_o**	0.7725*** (29.24)	1.0312** (2.27)	0.7857** (8.72)	1.1079** (2.11)	0.7739*** (28.28)	0.7737*** (29.46)
**Pop_d**	0.6990*** (23.64)	−1.8736*** (-3.60)	0.7122*** (30.42)	−1.7969*** (−2.93)	0.7034*** (22.36)	0.7034*** (26.78)
**Agdp_o**	−0.2946*** (−7.24)	0.2503 (1.31)	−0.11 (−2.82)	0.3771* (1.73)	−0.4009*** (−9.64)	−0.4036*** (−10.59)
**Agdp_d**	0.6282*** (15.80)	−0.2159 (-1.22)	0.8128*** (13.15)	−0.0891 (−0.42)	0.4822*** (12.09)	0.4761*** (12.39)
**Aenergy_o**	0.9686*** (19.77)	0.5357 (1.56)	0.9515** (9.31)	0.3928 (1.13)	0.9811*** (19.51)	0.9808*** (17.75)
**Aenergy_d**	0.0134 (0.23)	0.6151** (1.97)	−0.0037 (−0.05)	0.4721 (1.52)	0.0786 (1.29)	0.0828 (1.49)
**MarketSeg_od**	−2.0410*** (−3.88)	−0.5338 (−1.05)	−1.3552 (−0.67)	−0.3857 (−0.77)	−1.1004*** (−2.70)	−1.0703*** (−2.68)
**Distance_od**	−0.2730*** (−7.43)	3.9047** (2.26)	−0.2215* (−3.72)	4.0102** (2.28)	−0.3081*** (−8.13)	−0.3093*** (-8.32)
**Continguity_od**	0.1584*** (2.62)	7.4106** (2.50)	0.2340 (2.20)	7.6137** (2.53)	0.1077* (1.74)	0.1061* (1.69)
**Constant**	−15.2487*** (−25.44)	−22.1063* (−1.95)	−19.3009** (−29.48)	−26.4231** (−2.19)	−12.5549*** (−21.65)	−12.4610*** (−24.56)
**Observations**	2610	2610	2610	2610	2610	2610
**R-squared-overall**	0.6878	0.9090	0.7093	0.9096	0.6806	0.6801
**Province-time FE**	NO	YES	YES	YES	NO	NO
**Twoway FE**	NO	NO	NO	YES	NO	NO

Note: Superscript *** indicates significance at 1% level, superscript

** indicates significance at 5% level, and superscript

* indicates significance at 10% level.

#### 3.2.2 The effect of market segmentation on interprovincial CO_2_ embodied flow within Eastern China

To investigate further the impact of market segmentation on interprovincial embodied CO_2_ flow for different regions, we estimated the effect within different regions and between regions. We first investigated the effect within east China. The results are shown in Table 2. The regression model shows that market segmentation had a significant negative effect on the interprovincial embodied CO_2_ flow within eastern China, and the absolute values of the coefficients were larger than that for the whole country, showing a more critical impact of market segmentation on interprovincial embodied CO_2_ flow within eastern China than entire China. For independent variables, market segmentation also had a similar impact compared to the whole of China.

**Table 2:** The gravity estimated results using interprovincial embodied CO_2_ flows within Eastern China.

Variables	OLS	FE	FE-T	FE-TW	RE-GLS	RE-MLE
**Pop_o**	0.5413***	2.5830**	0.5570**	2.2950*	0.5484***	0.5484***
	(11.21)	(2.05)	(7.86)	(1.84)	(11.32)	(10.91)
**Pop_d**	0.6294***	0.2374	0.6451***	−0.0505	0.6280***	0.6279***
	(12.20)	(0.17)	(38.03)	(−0.03)	(12.24)	(12.48)
**Agdp_o**	−1.0292***	−0.9582	−0.8246**	−1.9509**	−1.1050***	−1.1055***
	(−9.78)	(−1.41)	(−5.81)	(−2.13)	(−10.95)	(−10.71)
**Agdp_d**	0.6238***	−1.3795*	0.8284**	−2.3722**	0.5030***	0.5019***
	(6.39)	(−1.84)	(6.15)	(−2.29)	(5.21)	(4.80)
**Aenergy_o**	1.7872***	3.0017***	1.7677**	3.8113***	1.8300***	1.8305***
	(11.45)	(2.63)	(4.86)	(2.97)	(11.19)	(11.57)
**Aenergy_d**	0.4280***	2.9307**	0.4085	3.7402**	0.5059***	0.5068***
	(2.74)	(2.15)	(0.92)	(2.43)	(3.02)	(3.19)
**Market_seg_od**	−4.6566***	−2.2883**	−4.7316*	−1.778	−3.5211***	−3.5144***
	(−4.30)	(−2.22)	(−3.01)	(−1.47)	(−3.84)	(−3.83)
**Distance_od**	−0.3593***	9.4592**	−0.2608**	10.0692**	−0.3886***	−0.3887***
	(−4.64)	(2.11)	(−7.68)	(2.48)	(−5.00)	(−4.80)
**Continguity_od**	0.0051	19.5858**	0.1260	21.0542**	−0.0334	−0.0336
	(0.04)	(2.20)	(1.35)	(2.37)	(−0.26)	(−0.23)
**Constant**	−5.5926***	−72.6412**	−0.4403***	−53.2841	−3.5040**	−3.4884***
	(−3.36)	(−2.20)	(−7.92)	(−1.58)	(−2.37)	(−2.58)
**Observations**	468	468	468	468	468	468
**R-squared-overall**	0.7506	0.9022	0.7612	0.9036	0.7479	0.7479
**Province-time FE**	NO	YES	YES	YES	NO	NO
**Twoway FE**	NO	NO	NO	YES	NO	NO

Note: Superscript *** indicates significance at 1% level, superscript

** indicates significance at 5% level, and superscript

* indicates significance at 10% level.

#### 3.2.3 The effect of market segmentation on interprovincial CO_2_ embodied flow within Central China

In Table 3, the result of the regression of Central China shows that the market segmentation had a negative effect on interprovincial CO_2_ flow within Central China, and the absolute of the coefficient was larger than that in Eastern China and the whole of China, which implicates that market segmentation was not significant. The population of origin and destination had an insignificant positive effect on the embodied CO_2_ flow.

**Table 3:** The gravity estimated results using interprovincial embodied CO_2_ flows within Central China.

Variables	OLS	FE	FE-T	FE-TW	RE-GLS	RE-MLE
**Pop_o**	0.9636***	5.6994	1.2745	3.8497	0.9209***	0.9077***
	(5.51)	(1.13)	(2.91)	(0.91)	(5.55)	(3.87)
**Pop_d**	0.3445	0.8337	0.6554**	−1.016	0.3932	0.4114*
	(1.35)	(0.24)	(5.39)	(−0.35)	(1.49)	(1.75)
**Agdp_o**	−0.8708	1.7154	−3.9739***	1.7079	−0.3857	−0.2165
	(−1.54)	(1.25)	(−24.81)	(1.01)	(−0.71)	(−0.39)
**Agdp_d**	1.0624	−0.6599	−2.0407**	−0.6673	0.6442	0.4918
	(1.67)	(−0.5)	(−5.83)	(−0.31)	(1.04)	(0.89)
**Aenergy_o**	1.1066***	−0.65	1.9637***	−1.4479	0.9696***	0.9174***
	(4.98)	(−0.29)	(16.65)	(−0.81)	(4.33)	(3.76)
**Aenergy_d**	−0.5216**	−1.1827	0.3355*	−1.9806	−0.4454*	−0.4199*
	(−2.06)	(−0.68)	(2.95)	(−1)	(−1.78)	(−1.77)
**Market_seg_od**	−8.4483	−14.4614	−4.9188	−11.6143	−8.398	−8.4242
	(−1.40)	(−1.27)	(−1.17)	(−1.05)	(−1.55)	(−1.18)
**Distance_od**	0.2546	−50.5848	−0.2011	−145.2234	0.278	0.2891
	(0.77)	(−0.31)	(−1.73)	(−1.02)	(0.86)	(0.89)
**Continguity_od**	0.3124	−42.7756	0.1297**	−115.8562	0.3208	0.3248
	(1.13)	(−0.34)	(7.1)	(−1.05)	(1.14)	(1.14)
**Constant**	−15.7286***	289.2368	40.2041**	980.5679	−16.5537***	−16.8132***
	(−3.63)	(0.24)	(5.51)	(0.98)	(−3.74)	(−4.44)
**Observations**	90	90	90	90	90	90
**R-squared-overall**	0.5587	0.8652	0.6843	0.8745	0.5529	0.5481
**Province-time FE**	NO	YES	YES	YES	NO	NO
**Twoway FE**	NO	NO	NO	YES	NO	NO

Note: Superscript *** indicates significance at 1% level, superscript

** indicates significance at 5% level, and superscript

* indicates significance at 10% level.

#### 3.2.4 The effect of market segmentation on interprovincial CO_2_ embodied flow within Western China

Western China is the least developed region in China. Table 4 reports the regression results of the gravity model of interprovincial embodied CO_2_ flows within Western China. The results show that market segmentation had no significant effect on the interprovincial embodied CO_2_ flows in western China, which was different from that of China and Eastern China, implicating that the embodied CO_2_ flows in Western China were more related to other factors rather than market segmentation.

**Table 4:** The gravity estimated results using interprovincial embodied CO_2_ flows within Western China.

Variables	OLS	FE	FE-T	FE-TW	RE-GLS	RE-MLE
**Pop_o**	0.8433***	2.5065	0.8324***	3.1355	0.7673***	0.7695***
	(7.57)	(1.11)	(20.34)	(1.38)	(6.12)	(7.23)
**Pop_d**	0.6972***	2.2822	0.6863**	2.9112	0.7259***	0.7254***
	(6.47)	(1.29)	(7.06)	(1.55)	(6.17)	(6.90)
**Agdp_o**	−0.2256	0.9387*	0.0231	1.2885**	−0.0942	−0.0987
	(-1.57)	(1.88)	(0.21)	(2.14)	(−0.60)	(−0.59)
**Agdp_d**	0.4585***	−0.0861	0.7072**	0.2637	0.2804**	0.2839*
	(3.28)	(−0.18)	(4.97)	(0.46)	(2.05)	(1.70)
**Aenergy_o**	0.8233***	−1.2829	0.7340**	−0.8276	0.6207***	0.6269***
	(4.17)	(−1.24)	(6.83)	(−0.73)	(2.81)	(2.82)
**Aenergy_d**	−0.279	0.0164	0.3682	0.4717	−0.1717	−0.1737
	(−1.45)	(0.02)	(−1.4)	(0.5)	(−0.87)	(−0.80)
**Market_seg_od**	−1.4249	1.9423	0.8929	2.0679	0.6082	0.5846
	(−0.89)	(1.25)	(−0.65)	(1.27)	(0.43)	(0.46)
**Distance_od**	−0.3109**	−1.1360**	0.3289**	−0.8208	−0.2831**	−0.2838***
	(−2.40)	(−2.25)	(−4.89)	(−2.28)	(−2.10)	(−2.60)
**Continguity_od**	0.2242	−2.7673**	0.2216	−2.9826**	0.2343	0.2339*
	(1.29)	(−2.17)	(1.66)	(−1.47)	(1.31)	(1.69)
**Constant**	−14.1404***	−39.2275	−18.3781***	−58.7102**	−13.4460***	−13.4499***
	(−10.40)	(−1.57)	(−22.68)	(−2.07)	(−10.06)	(−10.90)
**Observations**	330	330	330	330	330	330
**R-squared-overall**	0.6287	0.8732	0.6373	0.8743	0.6230	0.6232
**Province-time FE**	NO	YES	YES	YES	NO	NO
**Twoway FE**	NO	NO	NO	YES	NO	NO

Note: Superscript *** indicates significance at 1% level, superscript

** indicates significance at 5% level, and superscript

* indicates significance at 10% level.

#### 3.2.5 The effect of market segmentation on interprovincial CO_2_ embodied flow between Eastern and Central China

To investigate further the market segmentation’s impact on interprovincial embodied CO_2_ flow, we ran the regression of interprovincial flow further at the cross-region level.

First, we ran the gravity model between Eastern and Central China. The regression results are reported in Table 5. The results show that the market segmentation played a negative role in embodied CO_2_ flow and that it is significant at 10% confidence level in the OLS model and the random model, implicating that the more market segmentation it is, the less interprovincial embodied CO_2_ flow across Eastern and Central China. We can see that geographical distance had a significant negative effect on the embodied CO_2_ flows at the 1% level OLS model and the random model. The population of origin and destination had a significant positive impact on embodied CO_2_ flows, whereas the GDP per capita of origin and destination had opposite effects. The GDP per capita of origin is significantly negative, and the GDP per capita of GDP origin is significantly positive. These results are in accordance with the regression results at the national level.

**Table 5:** The gravity estimated results using interprovincial embodied CO_2_ flows between Eastern and Central China.

Variables	OLS	FE	FE-T	FE-TW	RE-GLS	RE-MLE
**Pop_o**	0.6107***	1.2749	0.6303**	1.5654	0.6116***	0.6116***
	(10.36)	(1.31)	(6.18)	(1.49)	(10.15)	(9.44)
**Pop_d**	0.7386***	−1.3724	0.7578***	−1.0875	0.7265***	0.7268***
	(9.56)	(−1.09)	(19.69)	(−0.78)	(9.08)	(11.23)
**Agdp_o**	−0.6694***	−0.1132	−0.3940*	0.4003	−0.7174***	−0.7172***
	(−8.24)	(−0.22)	(−3.24)	(0.69)	(−8.92)	(−8.37)
**Agdp_d**	0.7180***	0.0997	0.9922**	0.5980	0.6400***	0.6410***
	(9.20)	(0.21)	(5.01)	(1.11)	(8.66)	(7.48)
**Aenergy_o**	1.1527***	0.7020	1.1018***	0.3377	1.1531***	1.1533***
	(10.40)	(0.7)	(38.24)	(0.35)	(9.74)	(9.95)
**Aenergy_d**	−0.0331	0.4450	−0.0827**	0.0970	0.015	0.014
	(−0.30)	(0.43)	(−8.45)	(0.10)	(0.13)	(0.12)
**Market_seg_od**	−2.6870*	−1.5986	−2.3595	−0.8739	−2.0936*	−2.0979*
	(−1.95)	(−1.12)	(−1.59)	(−0.61)	(−1.90)	(−1.76)
**Distance_od**	−0.4316***	−3.1899	−0.3238**	−1.8324	−0.4516***	−0.4514***
	(−5.41)	(−0.46)	(−5.82)	(−0.17)	(−5.63)	(−5.44)
**Continguity_od**	0.1323	−1.5618	0.2471	−0.8511	0.1154	0.1154
	(1.03)	(−0.32)	(1.76)	(−0.26)	(0.89)	(0.82)
**Constant**	−10.3957***	20.4546	−16.7696*	−3.1785	−8.9249***	−8.9388***
	(−7.58)	(0.48)	(−4.04)	(−0.07)	(−7.11)	(−6.91)
**Observations**	468	468	468	468	468	468
**R-squared-overall**	0.7016	0.9087	0.7135	0.9114	0.7000	0.7000
**Province-time FE**	NO	YES	YES	YES	NO	NO
**Twoway FE**	NO	NO	NO	YES	NO	NO

Note: Superscript *** indicates significance at 1% level, superscript

** indicates significance at 5% level, and superscript

* indicates significance at 10% level.

#### 3.2.6 The effect of market segmentation on interprovincial CO_2_ embodied flow between Eastern and Western China

In this sub-section, we continue running the regression at cross regional interprovincial CO_2_ flow between Eastern and Western China. Table 6 reports the results, showing that the population of origin and destination and GDP per capita of origin and destination had the same effect as other analyzed cases. The market segmentation effect was not significant, implicating that market segmentation’s impact on interprovincial CO_2_ flow was unclear between Eastern and western China. Other variables, such as geographical distance and continuity, had significant negative and positive effects on interprovincial CO_2_ flow.

**Table 6:** The gravity estimated results using interprovincial embodied CO_2_ flows between Eastern and Western China.

Variables	OLS	FE	FE-T	FE-TW	RE-GLS	RE-MLE
**Pop_o**	0.8097***	−0.0926	0.8219***	−0.2216	0.7952***	0.7943***
	(20.76)	(−0.14)	(12.11)	(−0.29)	(18.89)	(18.14)
**Pop_d**	0.7426***	−3.2921***	0.7651***	−3.4307***	0.7404***	0.7400***
	(13.73)	(−3.74)	(64.42)	(−3.43)	(12.51)	(16.91)
**Agdp_o**	−0.1396**	0.1720	0.2361**	0.2257	−0.3324***	−0.3390***
	(−2.19)	(0.58)	(4.80)	(0.66)	(−5.33)	(−5.40)
**Agdp_d**	0.5954***	−0.1154	0.9547**	−0.1120	0.3703***	0.3619***
	(8.82)	(−0.41)	(6.39)	(−0.33)	(5.28)	(5.70)
**Aenergy_o**	0.9450***	0.7356	0.8365**	0.5386	0.9907***	0.9921***
	(11.66)	(1.45)	(9.08)	(1.02)	(11.9)	(9.78)
**Aenergy_d**	0.1131	0.7219	0.0296	0.5677	0.2480*	0.2559**
	(0.94)	(1.48)	(0.74)	(1.14)	(1.93)	(2.50)
**Market_seg_od**	−1.3033*	2.5922***	1.3939	2.0525*	1.1214*	1.2200*
	(−1.89)	(2.77)	(1.88)	(1.94)	(1.94)	(1.68)
**Distance_od**	−0.3059***	41.2580***	−0.2254*	43.1704***	−0.3537***	−0.3554***
	(−4.30)	(5.05)	(−3.34)	(4.37)	(−4.64)	(−4.87)
**Continguity_od**	0.4145***	10.3971***	0.4146	11.0753***	0.4070***	0.4061**
	(3.40)	(4.85)	(2.81)	(4.20)	(3.19)	(2.30)
**Constant**	−16.9483***	−269.7809***	−24.7611***	−281.5289***	−12.4261***	−12.2607***
	(−15.37)	(−5.06)	(−13.16)	(−4.68)	(−11.94)	(−12.14)
**Observations**	858	858	858	858	858	858
**R-squared-overall**	0.7034	0.9216	0.7387	0.9223	0.6919	0.6911
**Province-time FE**	NO	YES	YES	YES	NO	NO
**Twoway FE**	NO	NO	NO	YES	NO	NO

Note: Superscript *** indicates significance at 1% level, superscript

** indicates significance at 5% level, and superscript

* indicates significance at 10% level.

#### 3.2.7 The effect of market segmentation on interprovincial CO_2_ embodied flow between Central and Western China

The last case was interprovincial embodied CO_2_ flow between Central and Western China. Both regions were underdeveloped. Table 7 reports the regression results of this case. The results show that market segmentation had a significant negative impact on interprovincial embodied CO_2_ emissions. Other dependent variables, such as the population of origin and destination, had a significant positive effect on all estimation methods. The GDP per capita of origin and destination had a different impact on embodied CO_2_ flows. The energy consumption per capita of origin had a significant positive effect on embodied CO_2_ flows, whereas the destination’s energy consumption per capita had no significant effect. Geographical distance had a significant negative effect on the fixed model but not on OLS and random models.

**Table 7:** The gravity estimated results using interprovincial embodied CO_2_ flows between Central and Western China.

Variables	OLS	FE	FE-T	FE-TW	RE-GLS	RE-MLE
**Pop_o**	1.0398***	5.9155***	1.0353**	6.0073***	1.0344***	1.0345***
	(13.88)	(4.27)	(7.27)	(4.18)	(13.70)	(15.12)
**Pop_d**	0.7759***	0.4857	0.7713***	0.5775	0.7784***	0.7783***
	(11.06)	(0.32)	(23.57)	(0.36)	(11.22)	(11.38)
**Agdp_o**	−0.4194***	0.1481	0.2927*	0.4099	−0.3810***	−0.3831***
	(−3.17)	(0.46)	(−3.05)	(0.99)	(−3.10)	(−3.01)
**Agdp_d**	0.4386***	−0.1648	0.5653*	0.0971	0.3759***	0.3783***
	(3.45)	(−0.45)	(4.18)	(0.18)	(2.97)	(2.97)
**Aenergy_o**	1.0822***	0.9760**	1.0428***	0.8444*	1.0660***	1.0669***
	(9.56)	(2.18)	(11.18)	(1.80)	(9.53)	(9.00)
**Aenergy_d**	−0.0466	−0.2118	0.0860	−0.3434	−0.0311	−0.0315
	(−0.39)	(−0.40)	(−2.37)	(−0.63)	(−0.26)	(−0.27)
**Market_seg_od**	−2.5997***	0.0006	2.3943	−0.1753	−0.7514	−0.7837
	(−2.62)	(0.00)	(−1.89)	(−0.17)	(−0.91)	(−0.93)
**Distance_od**	−0.0986	−15.6018***	0.0948*	−15.6436***	−0.0962	−0.0963
	(−1.03)	(−3.42)	(−3.74)	(−3.45)	(−1.00)	(−0.99)
**Continguity_od**	0.2846**	−8.8846***	0.2684	−9.0466***	0.2975**	0.2973**
	(2.49)	(−4.11)	(2.09)	(−4.26)	(2.57)	(2.38)
**Constant**	−16.3074***	56.1644*	−18.5890***	50.2137	−16.0987***	−16.1013***
	(−14.47)	(1.72)	(−12.84)	(1.36)	(−14.44)	(−14.70)
**Observations**	396	396	396	396	396	396
**R-squared-overall**	0.7022	0.9025	0.7048	0.9033	0.6992	0.6993
**Province-time FE**	NO	YES	YES	YES	NO	NO
**Twoway FE**	NO	NO	NO	YES	NO	NO

Note: Superscript *** indicates significance at 1% level, superscript

** indicates significance at 5% level, and superscript

* indicates significance at 10% level.

From the above, the higher the degree of market segmentation is, the stronger the hindrance to interprovincial embodied carbon flow will be. And vice versa, the higher the degree of market integration is, the easier the carbon will flow. The underlying mechanism is that market integration facilitates the flow of goods and factors among regions, including carbon intensive products. The comparative advantage of backward regions in carbon intensive products is made full use of. High pollution industry is more likely to be transferred from developed regions to backward regions, resulting in more interprovincial carbon flows.

## 4. Conclusion and policy implications

### 4.1 Conclusions

As interprovincial embodied CO_2_ flows in China have been calculated and analyzed in many aspects. In this study, we recalculated the interprovincial embodied CO_2_ flows in 2007, 2010, and 2012 in China by using the latest MRIO table. We then sought to find how the interprovincial embodied CO_2_ flows were affected by market segmentation in China. By using the gravity model, we estimated the results at the national and within regional and cross-regional levels. The conclusions of this study are summarized as follows:

1. The total volume of interprovincial CO_2_ flow did not increase significantly from 2007 to 2012. However, the pattern of embodied CO_2_ flow changed considerably. Compared with 2007, the major flows were more concentrated in the eastern and central provinces.
2. Market segmentation significantly decreased the interprovincial embodied CO_2_ flows in China. Within the regional level, market segmentation had a significant negative effect on interprovincial embodied CO_2_ flows within Eastern China but not in Central and Western China. Between regional levels, market segmentation was not a significant hindrance to interprovincial embodied CO_2_ flows between Eastern and Central China and between Eastern and Western China. However, market segmentation had a significant negative impact on interprovincial embodied CO_2_ flows between Central and Western China.
3. Other variables, such as the population of origin and destination, had a significant positive impact on interprovincial embodied CO_2_ flows. The GDP per capita of origin and destination had a divergent effect on interprovincial embodied CO_2_ flows. The GDP per capita had a significant positive effect, whereas the GDP of origin showed a significant negative effect. Geographical distance had a significant negative impact on interprovincial embodied CO_2_ flows.

### 4.2 Policy implications

Since the opening up, China has been eliminating its market segmentation between provinces, achieving remarkable progress. Our results show that the process of eliminating market segmentation was accompanied by interprovincial embodied CO_2_ flows, especially from less developed provinces to developed provinces, raising more sharp environmental equality issues. Moreover, domestic carbon transfer could hardly help China reduce its overall carbon emission. Several relevant policies can be raised to deal with environmental equality issues caused by market segmentation elimination.

First, we recommend that China considers special ecological compensation in this process. For example, ecological compensation standards can be set according to the amount of interprovincial embodied CO2 flow or other pollutant flow, so as to reduce regional ecological environment imbalance caused by the market.

Second, China should speed up the construction of carbon trading market and promote the pilot work of carbon finance innovation. The construction of national carbon trading market will help to promote regional environment equity.

Third, given the composition effects resulting from interprovincial trade, stricter environmental regulations should be implemented within the whole country.

## Supporting information

S1 Appendix (DOCX)
