# Supplementary material for: Does market segmentation hinder interregional CO2 flow in China? — Evidence from China’s interprovincial MRIO table
Source: PLoS One. 2021 Aug 2;16(8):e0255518. doi: 10.1371/journal.pone.0255518 (PMC8328345; doi:10.1371/journal.pone.0255518)
Supplement: S1 Appendix — (DOCX) [file pone.0255518.s001.docx]

**S1 Appendix**

Table A1 Regional division of mainland China

| Regions | Provinces |
| --- | --- |
| Eastern China | Beijing, Tianjin, Hebei, Shanghai, Jiangsu, Zhejiang, Fujian, Shandong, Guangdong, Hainan, Liaoning, Jilin, and Heilongjiang |
| Central China | Shanxi, Anhui, Jiangxi, Henan, Hubei, and Hunan |
| Western China | Inner Mongolia, Guangxi, Chongqing, Sichuan, Guizhou, Yunnan, Tibet, Shannxi, Gansu, Qinghai, Ningxia, and Xinjiang. |
